# THE MEDIATING EFFECT OF SELF-TRANSCENDENCE IN THE RELATIONSHIP BETWEEN SOCIAL SUPPORT AND SUCCESSFUL AGING

**DOI:** 10.1093/geroni/igae098.2893

**Published:** 2024-12-31

**Authors:** Do Thi Thu Huyen, Moonkyoung Park, Xianqi Gao

**Affiliations:** Chungnam National University, Daejeon, Republic of Korea; Chungnam National University, Daejeon, Republic of Korea; Chungnam National University, Daejeon, Republic of Korea

## Abstract

**Background:**

The migration of old adults Chinese often involves accompanying their children to assist with caregiving for the younger generation, a strategy that addresses the shortfall in familial support.However, it remains unclear whether these old adults migrants can effectively adapt to their new environments, maintain their daily lives, and age successfully. Access to psychological and social resources is crucial for successful aging. Among these, transcendence is highlighted as a key psychological component. Purpose To verify the mediating effect of transcendence in the relationship between social support and successful aging among Chinese migrant old adults.

**Methods:**

Participants consisted of 306 Chinese migrant old adults aged 60 and above, and data on demographic information, social support, successful aging, and transcendence were collected using a structured questionnaire from August to December 2023. Analyzed using the SPSS 26.0 and AMOS 26.0 software programs. Results Social support was significantly correlated with successful aging and transcendence. Social support had a significant direct effect on both successful aging (β=.480, p<.001) and transcendence (β=.54, p<.001). Transcendence also had a significant direct effect on successful aging (β=.660, p<.001). The mediating effect of transcendence between social support and successful aging was significant (β=.306,p=.008,95%CI [.220-.392]).

**Conclusion:**

This study indicate that transcendence has a mediating effect on achieving successful aging among Chinese migrant old adults. Since social support can enhance the capacity for transcendence, it is necessary to develop interventions that support transcendence, enhanced by social support, to promote successful aging among Chinese migrant old adults in new environments.

